# The Kananaskis Wildfire Charter: a good start

**DOI:** 10.1038/s41467-026-70040-y

**Published:** 2026-03-17

**Authors:** Kapil Yadav, Abigail Croker, Adriana Ford, William Hayes, Yiannis Kountouris, James Millington, Jayalaxshmi Mistry, Monika Moreu Vicente, Ol Perkins, Kate Schreckenberg, Cathy Smith, Maximilian Stiefel, Michel Valette

**Affiliations:** 1 Department of Geography, Royal Holloway, University of London, Egham, UK; 2 Department of Geography, King’s College London, London, UK; 3 Leverhulme Centre for Wildfires, Environment and Society, London, UK; 4 Centre for BioComplexity, Princeton University, Princeton, NJ USA; 5 Centre for Environmental Policy, Imperial College London, London, UK

**Keywords:** Sustainability, Law, Fire ecology

## Abstract

The Kananaskis Wildfire Charter marks an important step toward addressing increasingly extreme wildfires. Attending to complex drivers, supporting Indigenous and local fire stewardship, and shifting toward integrated fire management will help ensure equitable, effective, and ecologically grounded governance and management.

The Kananaskis Wildfire Charter is one of the first international policy frameworks to outline shared commitments for addressing wildfire risk globally. Launched at the 2025 G7 Summit, it marks a timely and welcome step toward coordinated international action on wildfire. Its broad focus on preparedness, response, recovery, knowledge-sharing, and restoration is commendable, and its commitment to a ‘whole-of-society’ approach and recognition of Indigenous land management practices signal an important shift away from a ‘no-fire’ suppression paradigm.

Realising the Charter’s potential to catalyse transformative shifts in wildfire governance will depend on how its commitments are interpreted and implemented. We identify three areas that warrant further attention: (1) moving beyond an ignition-centred framing toward approaches that recognise the broader climate, ecological, and political drivers of wildfire; (2) clearer articulation of governance arrangements that support the autonomy and fire stewardship of Indigenous Peoples and local communities; and (3) shifting from predominantly reactive and technology-reliant approaches to proactive community-centred ones. Attending to these considerations can help ensure that the Charter contributes to equitable, effective, and ecologically grounded outcomes as it moves toward implementation.

We note the Charter’s emphasis on reducing human-caused ignitions, particularly accidental or ‘malicious’ ones. While this remains important, an emphasis on ignitions can inadvertently reinforce suppression-centric approaches and reproduce longstanding blame narratives that, in many regions, attribute wildfires to the burning practices of Indigenous Peoples and local communities. Fire regimes are being reshaped by complex, interlinked factors: climate change, land-use change, invasive species, altered ecologies, shifting rural economy, and weakened communal governance. For example, in the Brazilian Cerrado, more frequent and intense late-dry season wildfires over the past half century are linked to policies promoting agribusiness expansion, dispossession of Indigenous lands and longer fire seasons caused by climate change^1^. In Spain, declining rural economies and fuel buildup in abandoned grazing lands are driving larger and more frequent wildfires during climate-driven droughts. In such contexts, it becomes necessary to attend to these indirect but critical factors that shape fire regimes^2^.

The Charter’s emphasis on a ‘whole-of-society’ approach is a positive step, but it requires careful attention to governance arrangements and power relations. Experience across multiple regions shows that while Indigenous Peoples are often invited to contribute knowledge, decision-making authority and control remain concentrated in state institutions^3^. As the Kananaskis Charter moves into implementation, fire management informed by it should recognise and support the fire knowledge and expertise of both Indigenous and non-indigenous local rural communities. For Indigenous and local fire stewardship to be meaningful rather than symbolic, it requires secure land rights, recognition of Indigenous and local knowledge, and co-governance arrangements that provide decision-making power and responsibilities, as defined and led by communities. Without these conditions, there is a risk that fire stewardship becomes reduced to a standardised toolkit, in which specific Indigenous fire stewardship experiences—often drawn from settler-colonial contexts such as Australia or Canada—are generalised and extended elsewhere without due regard for local Indigenous sovereignty, kinship relations, or socio-ecological contexts^4^.

A related consideration is how fire is categorised and understood^5^. Without clear distinctions between intentional, well-managed, and often regenerative burning practices and destructive wildfires, there is a risk that all fire will be treated negatively in policy and practice. Across many regions, fire use by Indigenous Peoples and local communities is central to livelihoods, cultural practice and landscape management^3,6^. However, when these practices are criminalised, they undermine collective burning and knowledge sharing. In such contexts, burning often becomes more individualised and secretive, making it harder to manage safely and increasing the risk of escaped wildfire^6,7^.

When it comes to responding to wildfires, the Charter leans heavily on technical solutions such as early detection, forecasting, and firefighting. These tools are important and will continue to play a role in reducing wildfire risk. At the same time, experience from fire-prone regions suggests that a predominantly technology-focused reactive approach can limit investment in building capacity at the community level. As the Charter recognises, emergency response alone will not reduce long-term risk. Translating this recognition into practice will require integrated fire management approaches that emphasise wildfire prevention, response, and recovery through proactive, participatory, and locally grounded strategies^8^.

Finally, the broader paradigm of living with fire needs greater emphasis^9^. Resilience cannot be limited to infrastructure or rapid response. It must involve adapting how and where we live, recognising fire as an ecological and unavoidable process in fire-prone ecosystems, and strengthening community-owned and community-led approaches to fire management.

At the recently concluded COP30, the Call to Action on Integrated Fire Management and Wildfire Resilience, endorsed by 50 countries, reiterates and expands many of the commitments first articulated in the Kananaskis Wildfire Charter. As the Charter moves from words to action, we hope it becomes not just a tool for international coordination but a catalyst for transformation in fire governance. Achieving this requires centring justice, supporting diverse ways of living with fire, and recognising that resilience depends on rethinking both risk and response.
